# Prevalence of canid herpesvirus-1 infection in stillborn and dead neonatal puppies in Denmark

**DOI:** 10.1186/s13028-014-0092-9

**Published:** 2015-01-08

**Authors:** Rikke W Larsen, Matti Kiupel, Hans-Jörg Balzer, Jørgen S Agerholm

**Affiliations:** Section for Veterinary Reproduction and Obstetrics, Department of Large Animal Sciences, Faculty of Health and Medical Sciences, University of Copenhagen, Dyrlægevej 68, Frederiksberg, DK-1870 Denmark; Department of Pathobiology and Diagnostic Investigation, Diagnostic Center for Population and Animal Health Michigan State University, Lansing, MI 48910 USA; Vet Med Labor GmbH, Division of IDEXX Laboratories, Ludwigsburg, D-71636 Germany

**Keywords:** Canid herpesvirus-1, CaHV-1, Viral infection, Neonatal, Puppies, PCR, In situ hybridization

## Abstract

**Background:**

Canid herpesvirus-1 (CaHV-1) infection in puppies less than three weeks of age is often reported to be associated with a lethal generalized necrotizing inflammation and since the discovery of the virus in 1965 several reports of neonatal infections have been published. However, the significance of CaHV-1 for peri- and neonatal mortality in puppies remains unclear. Therefore, we examined stillborn and dead neonatal puppies in Denmark to determine the prevalence of infection and further to correlate infection levels with necropsy findings to assess the possible significance of the infection.

**Results:**

From a cross-sectional study of 57 dead puppies, 22.8% (n = 13) were confirmed positive for CaHV-1 by real-time polymerase chain reaction (PCR) of tissue pools of lung/liver and/or spleen/kidney. Specimens from PCR positive cases were further investigated by histology and in situ hybridization (ISH). High levels of CaHV-1 DNA were present in only one case in which lesions and ISH staining consistent with CaHV-1 infection were found as well. CaHV-1 concentrations in the other cases were low and a range of lesions not consistent with CaHV-1 were found. Similar, ISH staining was mostly negative in these except for one case with a few positive cells.

**Conclusion:**

CaHV-1 infection in stillborn and dead neonatal puppies in Denmark seems to be common, but the direct significance for puppy mortality remains unclear as only one of 13 PCR positive puppies (7.7%) had pathognomonic lesions.

**Electronic supplementary material:**

The online version of this article (doi:10.1186/s13028-014-0092-9) contains supplementary material, which is available to authorized users.

## Background

Canid herpesvirus-1 (CaHV-1) is an enveloped double stranded DNA virus belonging to the family *Herpesviridae*, subfamily *Alphaherpesviridae*, genus *Varicellovirus* [1]. CaHV-1 is phylogenetically related to α-herpesviruses affecting other animal species but surface receptors limit the host range of CaHV-1 to domestic dogs and other *Canidae* [2]. CaHV-1 was first reported by Carmichael *et al*. [3] as a cytopathic agent associated with a systemic fatal hemorrhagic infection in neonatal puppies by causing focal necrosis of parenchymal organs. The infection is usually asymptomatic or restricted to the upper airways or the genital tract in adult dogs, although infection of pregnant bitches may cause embryonic loss, abortion and stillbirth [3-6]. The immature status of newborn puppies makes them vulnerable to a systemic CaHV-1 infection as resistance seems to be correlated to the maturity of the immune system and thermoregulation [7,8].

Newborn puppies are usually suspected to have acquired the infection from oronasal secretions of the bitch, infected littermates or from other dogs in the household [9,2] though neonatal disease may also occur following intrauterine infection or from viral infection during passage through the birth canal [4-6].

The neonatal mortality rate in dogs is generally considered high although only a few studies have been published. Indrebø *et al*. [10] emphasized that the canine mortality within the first eight weeks of life may reach 17-30%. Other studies have identified bacterial infections and fetal asphyxia as the main causes of puppy mortality [11,12]. Reports of fatal neonatal CaHV-1 infections have been published [13,14] and even though serological studies from many European countries indicate that CaHV-1 infection is widespread in adult dogs, with antibody prevalence ranging from 40-88% [14-18] CaHV-1 is not considered to be a major cause of mortality in puppies [10-12]. However, information on the prevalence of CaHV-1 infection in neonatal dogs is sparse.

The aim of this study was to determine the prevalence of CaHV-1 infection in stillborn puppies and puppies dying within the first three weeks of life in Denmark and further to access the significance of the infection based on post mortem findings.

## Material and methods

### Study design, sampling and necropsy

The study was performed as a cross-sectional necropsy study from September 2012 to April 2013 based on stillborn puppies and puppies that died or were euthanized during their first three weeks of life. Information about the project was sent to 300 companion animal clinics throughout Denmark and also advertised on the internet. Breeders and veterinarians were asked to keep the dead puppies at 5°C until shipment, which should be done cooled. The submission was accompanied with a form including historical data such as breed, litter size, litter mortality, clinical signs and vaccination status for CaHV-1. Fifty-seven puppies originating from 37 litters and being of 26 different breeds formed the overall study population (see Additional file 1). Twenty-one cases were stillborn, 25 cases were aged from 1 to 7 days and 11 cases were older than 7 days. The age of puppy no. 33 was unknown as the submission was not accompanied by a form but it was estimated to a few days old based on the developmental stage. The submitted puppies originated from most parts of Denmark (see Additional file 2).

The puppies were necropsied upon arrival at the University and specimens were taken for histology and real-time polymerase chain reaction (PCR). Two tissue pools were sampled for PCR, i.e. liver/lung and spleen/kidney pools, when available and stored at −70°C. The time between death and necropsy varied from hours to days.

Specimens for histology and in situ hybridization (ISH) were taken from brain, heart, lung, liver, spleen, kidney and adrenal gland if tissues were available/suitable and fixed in 10% neutral buffered formalin, processed by routine methods, sectioned at 3 μm and stained with hematoxylin and eosin.

### Real-time PCR

Quantification of CaHV-1 DNA was done by a semi quantitative approach applying a real-time PCR assay at IDEXX Vet Med Laboratories, Ludwigsburg, Germany targeting a 116 bp fragment of the CaHV-1 DNA polymerase gene.

Samples were shipped overnight on dry ice to the laboratory where about 10 mg of each organ in a pool was comminuted and mixed with 180 μl ATL (QIAGEN, Hilden, Germany) and 20 μl Proteinase K solution (QIAGEN). Total nucleic acid was extracted from the tissues by using the QIAamp DNA Mini Kit (QIAGEN) according to the manufacturer’s instructions. PCR was performed using the LightCycler 480 system (Roche Diagnostics, Mannheim, Germany) with proprietary forward and reverse primers and a hydrolysis probe. The PCR was run with five quality controls, including a 1) PCR positive control, 2) PCR negative control, 3) negative extraction controls, 4) internal positive control (IPC) spiked into the lysis solution to monitor the nucleic acid extraction efficiency and presence or absence of inhibitory substance and 5) an environmental contamination monitoring control.

Samples were considered positive if the amplification exceeded the threshold within 45 repeated cycles. Results were given as crossing point values (Cp, the point at which the fluorescence of a sample rises above the background fluorescence) of undiluted, 1:10 and 1:100 dilutions (See Additional file 3). Samples with Cp >40 (flagged as “Late Cp call” (last five cycles) with a higher uncertainty by the LightCycler 480 software) were repeated in duplicate. If the Cp value could be reproduced in repeated analysis, the sample was considered positive, though indicative of a minimal amount of total viral DNA. The crossing points were calculated applying the Second Derivative Maximum method to standardize Cp calling.

As a sample’s Cp depends on the initial concentration of DNA in the sample the correlation between DNA concentration and Cp is inversed: A sample with a lower initial concentration of target DNA requires more amplification cycles to reach the Cp and a sample with higher concentration requires fewer cycles.

### In situ hydridization

In situ hybridization for CaHV-1 was performed on serial sections of formalin-fixed, paraffin-embedded tissues using an automated slide-processing system as previously described [19]. Briefly, sections of the selected tissue samples were prepared at 5 μm thickness and placed on positively charged slides. These slides were then submitted to deparaffinization and fixation using the Discovery XT automated slide processing system (Ventana Medical Systems, Inc., Tucson, AZ), as programed in the protocol for RiboMap ISH reagent system (Ventana Medical Systems). Proteolytic treatment was performed using Protease 3 (0.02 units/ml alkaline protease, Ventana Medical Systems) for 12 min at 37°C. Thereafter, the slides received pre-treatment through standard cell conditioning using the citrate buffer-based RiboCC reagent (Ventana Medical Systems) for 4 min at 95°C. The slides were then submitted to denaturation for 4 min at 95°C, followed by hybridization with a digoxigenin-labeled oligonucleotide probe (DIG-5′–GATATTGTGCAAGGTATATACTCAGTACAAGACCGGAAGCTGC–3′) that targets a highly conserved region of the TK gene sequence of CaHV-1 (nucleotide positions 330–372, GenBank Acc. No. D83054) suspended in hybridization buffer (RiboHybe, Ventana Medical Systems). The time of hybridization was 2 h at 42°C. The concentration used for the generic probe was 682 ng/ml (dilution 1:1,000). For the generic probe, three stringency washing steps were performed using 2.0× RiboWash (Ventana Medical Systems; equivalent to 2.0× saline sodium citrate) each for 4 min at 52°C. After the stringency washes, the slides were incubated with anti-digoxigenin antibody for 32 min at 37°C. The anti-digoxigenin antibody was a rabbit monoclonal anti-digoxigenin antibody (Invitrogen Corporation, Frederick, MD) used at the dilution of 1:10,000. After streptavidin-alkaline phosphatase conjugate (UMap anti-Rb AP, Ventana Medical Systems) incubation for 24 min at 37°C, the signal was detected automatically using the BlueMap NBT/BCIP substrate kit (Ventana Medical Systems) for 44 min at 37°C. Finally, the sections were counterstained with the nuclear fast red equivalent reagent Red Counterstain II (Ventana Medical Systems) for 4 min before coverslipping. Tissue sections of a young puppy naturally infected with CaHV-1 were used as a positive control. For the negative reagent controls, sections were treated only with RiboHybe hybridization buffer.

### Histopathology

Tissue sections from PCR positive cases were examined and the most significant lesions recorded (Table 1). CaHV-1 was considered as the cause of death if pathognomonic multifocal coagulation necrosis were observed.

**Table 1:** **Findings in 13 puppies from 11 litters found positive for canid herpesvirus-1 by real-time polymerase chain reaction (PCR)**

**Litter no.**	**Puppy no.**	**Time of death (Days post partum)**	**PCR (Cp value)**		**Most significant lesions**	**In situ hybridization**
			**Liver/lung**	**Spleen/kidney**		
1	1	13	13.3	13.9	Acute disseminated necrosis in multiple tissues with intranuclear inclusion bodies	Strong labeling was detected in nuclei of cells in close proximity to focal areas of necrosis including hepatocytes, adrenal cortical cells, renal tubular epithelial cells, bronchial epithelial cells and pneumocytes.
2	2	12	29.5	31.4	Focal liver abscess. Mild macrocytic aspiration bronchopneumonia	A few ISH positive cells, most suggestive of macrophages based on morphology, were found in the liver, spleen and kidney
3	3	2	32.8	32.6	Mild macrocytic aspiration bronchopneumonia. Severe hepatic congestion.	Negative
	4	3	32.9	32.5	Mild interstitial suppurative hepatitis	Negative
4	7	12	37.8	36.7	Hepatomegaly. Widespread splenic single cell necrosis	Negative
5	8	0	34.0	NA	Severe autolysis. Inconclusive	ND
6	11	17	Negative	>40*	Hepatomegaly, splenomegaly and renomegaly. Alveolar hemorrhage and erythrophagocytosis	Negative
7	12	0	>40	39.4	Severe autolysis. Inconclusive	ND
	13	0	34.8	36.9	Severe autolysis. Inconclusive	Negative
8	15	0	35.7	>40	Polydactyli. Congenital atelectasis. Mild aspiration bronchopneumonia	Negative
9	16	14	38.9	Negative	Acute severe necrotizing and suppurative bronchopneumonia. Hepatomegaly and splenomegaly.	Negative
10	17	1	33.6	> 40	Mild macrocytic bronchopneumonia	Negative
11	18	14	31.3	Negative	Acute severe suppurative bronchopneumonia. Widespread acute suppurative interstitial hepatitis. Hepatomegaly, splenomegaly and renomegaly. Follicular purulent dermatitis.	Negative

Crossing point (Cp) values based on analysis of 1:10 dilutions of two tissue pools (liver/lung and spleen/kidney, respectively) see Additional file 3 for details.

NA: Tissues not available – probably eaten by the bitch. ND: Not done.

*Cp > 40 was considered positive though indicating a minimal amount of nuclei acid.

## Results

Fifty-seven puppies from 37 different litters formed the overall study population. The number of dead puppies per litter varied from one to eight, but submission of all dead puppies was not achieved in all cases (see Additional file 1). Analysis and statistical comparisons of historical data and potential risk factors e.g. litter size, clinical signs and litter mortality was inconclusive due to the low number of puppies (data not shown).

PCR analysis identified 13 puppies as being infected by CaHV-1 (22.8%). In the group of puppies older than 7 days, six puppies (54.5 %) were positive for CaHV-1 DNA. Furthermore, three of 25 cases (12.0 %) aged from 1 to 7-days-old and four of 21 stillborn puppies (19.0 %) had detectable levels of CaHV-1 DNA in the organs.

One case (Puppy 1) had high levels of CaHV-1 DNA in both tissue pools, while the other puppies had low levels with Cp values mostly above 30. CaHV-1 DNA amounts in the two tissue pools (lung/liver and spleen/kidney) were usually at the same level although some cases were negative in one pool and positive in the other (Table 1).

Puppy no. 19 tested negative for CaHV-1 in the tissue pools but the heart that was tested by a mistake was found positive in two independent DNA isolations (1^st^ preparation Cp (1:10) = 38.2 and 2^nd^ preparation Cp (undiluted) > 40). see Additional file 3 for details. The puppy was not included in the test positive group as this group was defined as puppies being positive in lung/liver and/or spleen/kidney.

Necropsy and subsequent histological examination of tissues from PCR positive puppies revealed a wide range of lesions of which the most significant are reported in Table 1. Three stillborn puppies (8, 12 and 13) had severe autolysis due to intrauterine death and retention. This compromised gross examination and hindered histology. Almost complete sets of tissues were available in the other cases although they displayed a certain degree of autolysis. Parts of the cranial mesenterial ganglion were included in the sections of the adrenal gland in seven cases.

**Figure 1:**
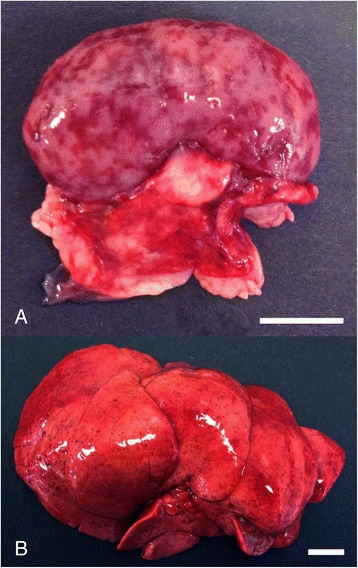
**Canid herpesvirus-1 associated lesions in the kidney and liver. A)** The kidney is enlarged and the cortex and serosa have widespread irregular confluent hemorrhages. **B)** The liver is enlarged with petechial hemorrhages. Bar = 1 cm. Puppy no. 1.

**Figure 2:**
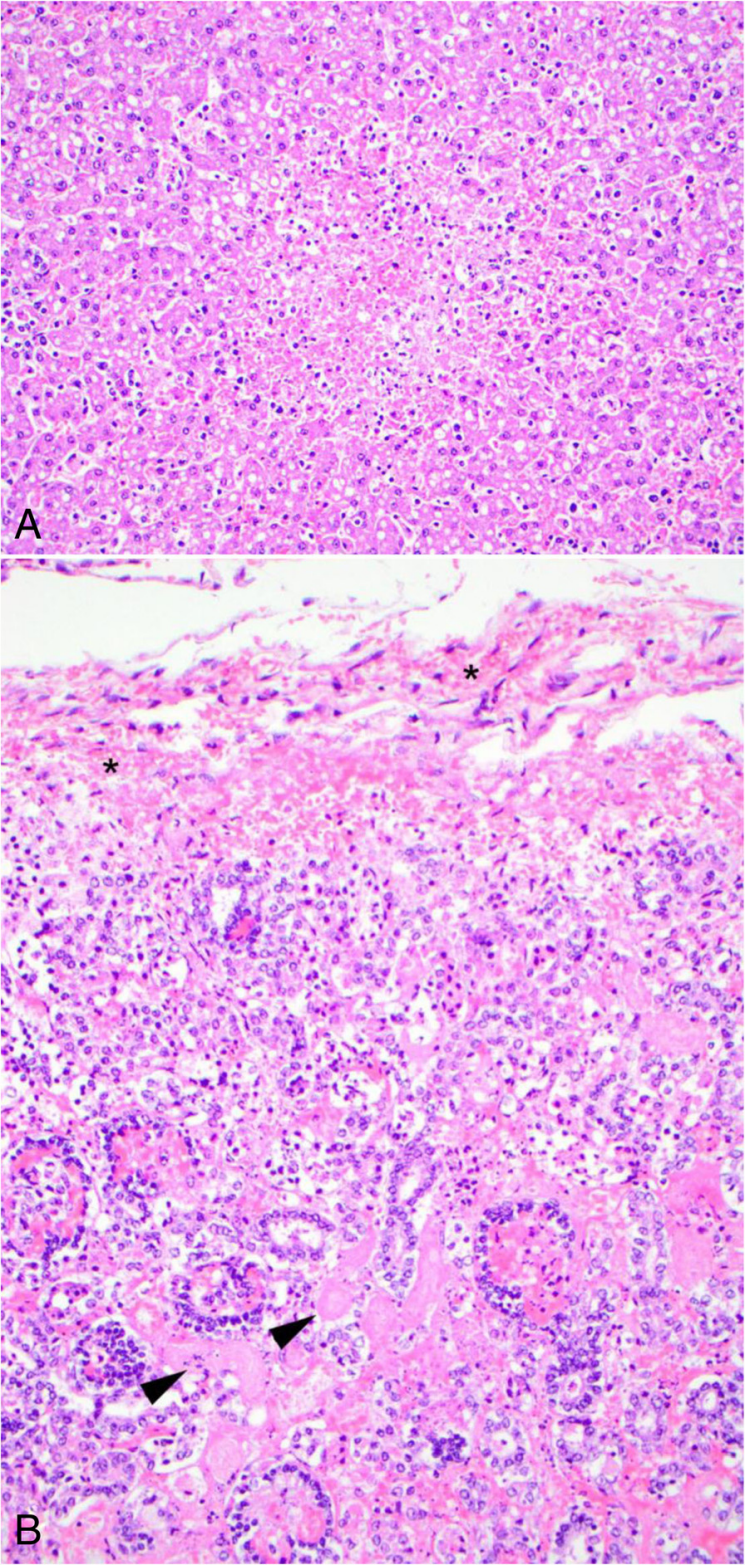
**Microphotographs of canid herpesvirus-1 associated lesions in the liver and kidney. A)** Acute focal necrosis of the liver parenchyma with central hemorrhage. **B)** Acute tubular necrosis (arrowheads) in the renal cortex. Grossly visible hemorrhages are present in the superficial cortex and serosa (asterisk). Hematoxylin and eosin. Puppy no. 1

**Figure 3:**
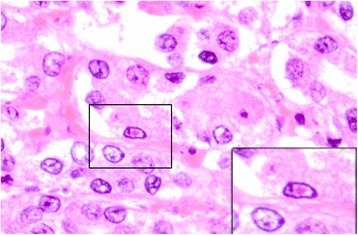
**Eosinophilic intranuclear inclusion body.** Acute tubular necrosis in the renal cortex with a canid herpesvirus-1 intranuclear inclusion body (insert). Hematoxylin and eosin. Kidney. Puppy no. 1.

Only puppy no. 1 had lesions consistent with a fulminant CaHV-1 infection, i.e. hepatomegaly, splenomegaly and bilateral renomegaly associated with widespread hemorrhages (Figure 1) that was histologically confirmed as being associated with acute disseminated necrosis in the lung, myocardium, liver, kidney and adrenal gland (Figure 2A,B) with presence of intranuclear eosinophilic inclusions bodies (Figure 3).

**Figure 4:**
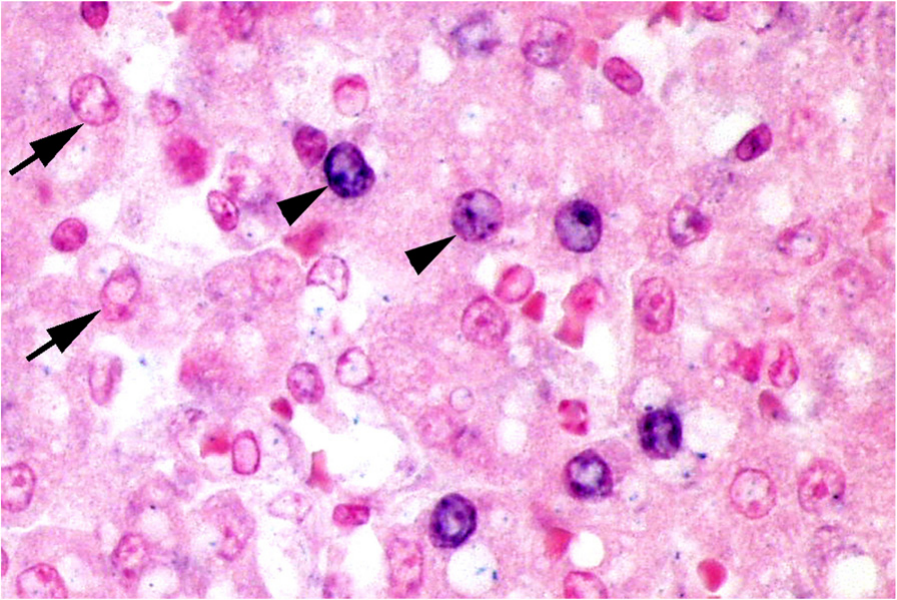
**Intranuclear canid herpesvirus-1 (CaHV-1) DNA labeled with in situ hybridization.** Multiple hepatocytes have CaHV-1 positive nuclei (arrowheads) seen as blue labeling. Examples of negative nuclei just counter stained by nuclear fast red are indicated by arrows. Liver. Puppy no. 1.

Significant ISH staining was only found in puppy no. 1 (Figure 4). Strong labeling was detected in nuclei of cells in close proximity to focal areas of necrosis including hepatocytes, adrenal cortical cells, renal tubular epithelial cells, bronchial epithelial cells and pneumocytes. In areas of necrosis and inflammation, positive signal was also detected in nuclei of endothelial cells and macrophages. A few ISH positive cells, most suggestive of being macrophages based on morphology, were found in the liver, spleen and kidney of puppy 2. The remaining puppies were negative (Table 1).

Based on the historical information obtained from the breeders, only one bitch (litter 3) had been vaccinated against CaHV-1. This was done as a two dose regime during pregnancy according to the manufacture’s protocol. Three of 10 puppies from her litter died during the neonatal period and were submitted. Two of the puppies were positive by PCR (puppy nos. 3 and 4) (Table 1). No interference of bitch vaccination and detection of CaHV-1 DNA in puppies are to be expected as the vaccine contains glycoproteins of the virus that is not transmitted in utero/transplacental. These three puppies had nursing problems during their first 24 h of life and probably received sparse amounts of colostrum.

## Discussion

CaHV-1 infection was found in puppies originating from most areas of Denmark and puppies of different breeds were affected. Although it is difficult to extrapolate from a study based on examination of 57 dead puppies from 37 litters to the Danish dog population in general, these findings still indicate that CaHV-1 occurs widely in the Danish dog population similarly as previously reported from other North European dog populations [14-18]. The overall prevalence of CaHV-1 infection in this study was 22.8%, but it varied between age groups. Although not statistically significant, the infection was most prevalent in puppies older than seven days (54.5%) while 19.0% and 12.0% of stillborn and puppies aged 1 to 7 days were PCR positive, respectively (see Additional file 1). This probably reflects factors such as an age dependent exposure risk and viral incubation period. Congenital infections seem to be rather common as 19.0% of the stillborn puppies were PCR positive and might reflect the possibility for the virus to be transmitted to the fetus in utero. Transplacental transmission of CaHV-1 has previously been shown to cause stillbirth in experimentally inoculated pregnant bitches [4,5]. Our study, however, could not directly correlate the cause of death in the stillborn puppies with CaHV-1 infection.

Only one puppy (no. 1) had lesions consistent with a fulminant CaHV-1 infection. These lesions correlated well with a high infection level and positive ISH labeling (Table 1). The significance of the infection for the other 12 puppies remains speculative due to the absence of pathognomic lesions and low infection levels. As the study was performed as a cross sectional necropsy study of spontaneously diseased puppies, a wide range of conditions have probably influenced the results, which is a limitation of such a study type. Exposure dose and time, stage of infection, colostrum intake, colostrum antibody levels, concomitant diseases and environmental influences are just some factors that may affect the course of infection and these factors were unknown in the present study.

Some of the puppies had lesions consistent with a bacterial disease, i.e. suppurative inflammation, while others had lesions indicative of intrauterine stress, such as congenital pneumonia associated with aspiration of amniotic epithelial cells. Infection with CaHV-1 may have predisposed puppies to other infectious causes or aggravated their course. Some puppies surviving an expected lethal oronasal challenge with a virulent CaHV-1 strain at three days of age had PCR positive lung and kidney samples 24 days post inoculation [20]. Inoculated puppies were small and underweight, with lesions of subacute interstitial pneumonia and subacute interstitial nephritis. Similar to the results presented here, characteristic CaHV-1 lesions were absent in the organs examined microscopically [20]. These data further support the hypothesis that a sublethal infection with CaHV-1 may predispose affected puppies to secondary bacterial infections. Widespread herpesviral infection has also been detected by PCR in non-canine species without associated characteristic lesions [21]. In one study, 6 of 10 seal pups were positive for phocine herpesvirus-1 (PhHV-1) but did not have histopathological evidence of characteristic viral lesions [21]. One premature animal died of a systemic PhHV-1 infection and three others had varying degrees of necrosis in the adrenal glands and liver, though died of other courses. Other positive cases without characteristic lesions were considered to have recovered from a clinical infection with detectable antibodies against the virus [21]. This phenomenon may explain at least some of the PCR findings in the present study, particularly in the older puppies which may have experienced a sublethal infection or may have recovered from a clinical infection but died of other courses or were euthanized due to weakness.

## Conclusion

CaHV-1 infection was diagnosed in 22.8% of 57 puppies that died or were euthanized during the first three weeks of life thus indicating that CaHV-1 infection is common in Danish puppies. Presence of viral DNA was more prevalent than characteristic CaHV-1 lesions and only one puppy was diagnosed with a systemic CaHV-1 infection.

## Additional files

Additional file 1:
**Breed, gender, litter mortality rate and overall polymerase chain reaction (PCR) results for canid herpesvirus-1 (positive (+)/negative (−)) in 57 puppies from 37 litter submitted for necropsy.**
Additional file 2:
**Map showing the geographical origin of puppies (litters) submitted for examination for canid herpesvirus-1 in Denmark, September 2012 to April 2013.**
Additional file 3:
**Real-time polymerase chain reaction assay for canid herpesvirus-1.** Results of crossing point values (Cp) of undiluted, 1:10 and 1:100 dilutions.
