# Supplementary material for: Prevalence of canid herpesvirus-1 infection in stillborn and dead neonatal puppies in Denmark
Source: Acta Vet Scand. 2015 Jan 8;57(1):1. doi: 10.1186/s13028-014-0092-9 (PMC4296690; doi:10.1186/s13028-014-0092-9)
Supplement: Additional file 1: — Breed, gender, litter mortality rate and overall polymerase chain reaction (PCR) results for canid herpesvirus-1 (positive (+)/negative (−)) in 57 puppies from 37 litter submitted for necropsy. [file 13028_2014_92_MOESM1_ESM.pdf]

Additional file 1

Breed, gender, litter mortality rate and overall polymerase chain reaction (PCR) result for canid herpesvirus-1 (positive (+)/negative (-)) in 57 puppies from 37 litter submitted for necropsy.

Litter no.	Puppy no.	Breed	Gender	Death (Days postpartum)	No. of dead puppies/ Litter size	PCR result* (+/-)
1	1	Boxer	M	13	8/9	+
2	2	Jack Russell	F	12[E]	1/3	+
3	3	Labrador	M	2	3/10	+
	4	Labrador	M	3		+
	5	Labrador	M	4		-
4	6	Jack Russell	M	5	5/6	-
	7	Jack Russell	M	12		+
5	8	Retriever cross	F	0	2/2	+
	9	Retriever cross	F	0		-
6	10	Pug	F	17[E]	2/2	-
	11	Pug	F	17[E]		+
7	12	French Bulldog	F	0	3/8	+
	13	French Bulldog	F	0		+
	14	French Bulldog	F	0		-
8	15	Norwegian Elkhound	M	0	1/3	+
9	16	Bernese Mountain	F	14[E]	2/11	+
10	17	Rottweiler	M	1	2/8	+
11	18	Grand Danoise	M	14	3/13	+
12	19	Afghan Hound	F	6	6/10	-
13	20	Terrier cross	F	0	1/3	-
14	21	Finnish Lapphund	F	0	1/7	-
15	22	Chihuahua	M	0	1/4	-
16	23	St. Bernard	M	0	3/4	-
	24	St. Bernard	F	0		-
	25	St. Bernard	F	0		-
17	26	Jack Russell	F	0	2/3	-
18	27	Golden Retriever	M	6	6/7	-
	28	Golden Retriever	F	4		-
	29	Golden Retriever	F	4		-
	30	Golden Retriever	F	4		-
19	31	Dachshund	F	3	2/3	-
	32	Dachshund	F	0		-
20	33	Unknown	F	?**	?	-
21	34	Scottish Terrier	F	10[E]	4/6	-
22	35	Chihuahua	M	0	2/4	-
23	36	German Shepherd	M	2	4/10	-
	37	German Shepherd	F	2		-
	38	German Shepherd	M	3		-

	39	German Shepherd	M	4		-
24	40	Basset Hound	F	4	3/8	-
25	41	Beagle	M	5	2/7	-
	42	Beagle	M	6		-
26	43	Danish Mastiff	F	1	2/12	-
27	44	Coton De Tulear	M	8[E]	2/4	-
28	45	Shih Tzu	F	5	1/6	-
29	46	Chihuahua	M	10	4/5	-
30	47	Chihuahua	M	4	1/5	-
31	48	French Bulldog	F	0	2/6	-
32	49	Jack Russell	F	0	3/3	-
	50	Jack Russell	F	0		-
	51	Jack Russell	F	0		-
33	52	Boxer	F	6	1/6	-
34	53	Newfoundland	M	0	1/10	-
35	54	Rottweiler	F	13	7/7	-
36	55	Boxer/Labrador cross	F	0	1/1	-
37	56	German Shepherd	F	2	5/8	-
	57	German Shepherd	M	2		-

*The PCR analysis was considered positive if one or both of the tissue pools (liver/lung and spleen/kidney) had a crossing point (Cp) value below 40.

** Estimated to a few days old (1-7 days) based on the developmental stage

E: Euthanized
