# Supplementary material for: Prevalence of canid herpesvirus-1 infection in stillborn and dead neonatal puppies in Denmark
Source: Acta Vet Scand. 2015 Jan 8;57(1):1. doi: 10.1186/s13028-014-0092-9 (PMC4296690; doi:10.1186/s13028-014-0092-9)

Additional file 2

Map showing the geographical origin for puppies (litters) submitted for examination for canid herpesvirus-1 in Denmark, September 2012 to April 2013.

Origin of litters with at least one puppy tested positive for CaHV-1 by polymerase chain reaction (PCR) are indicated by purple dots, while PCR negative litters are shown by blue dots.

It can be seen from the map that puppies were submitted from most parts of Denmark and the PCR positive litters seem to be randomly distributed.

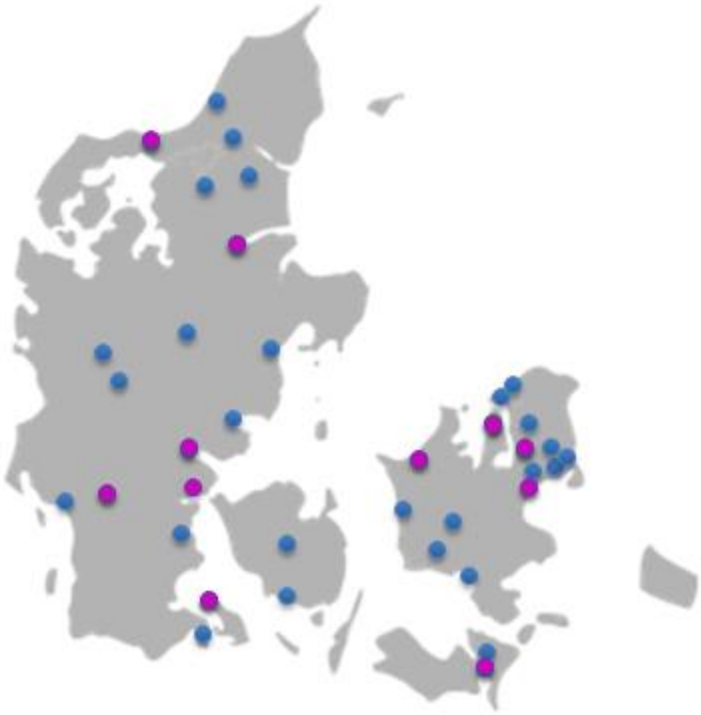

Supplement: Additional file 2: — Map showing the geographical origin of puppies (litters) submitted for examination for canid herpesvirus-1 in Denmark, September 2012 to April 2013. [file 13028_2014_92_MOESM2_ESM.pdf]
