# Supplementary material for: Prevalence of canid herpesvirus-1 infection in stillborn and dead neonatal puppies in Denmark
Source: Acta Vet Scand. 2015 Jan 8;57(1):1. doi: 10.1186/s13028-014-0092-9 (PMC4296690; doi:10.1186/s13028-014-0092-9)
Supplement: Additional file 3: — Real-time polymerase chain reaction assay for canid herpesvirus-1. Results of crossing point values (Cp) of undiluted, 1:10 and 1:100 dilutions. [file 13028_2014_92_MOESM3_ESM.pdf]

Additional file 3

Real-time polymerase chain reaction assay for canid herpesvirus-1. Results of crossing point values (Cp) of undiluted, 1:10 and 1:100 dilutions.

Puppy no.	Dilutions	PCR (Cp value)		Additional tissue analyzed	Remarks
		Liver/lung	Spleen/kidney		
1	Undiluted	16.0	16.6		
1	1:10	13.3	13.9		Positive
1	1:100	16.8	17.3		
2	Undiluted	39.4	>40*		
2	1:10	29.5	31.4		Positive
2	1:100	33.7	34.9		
3	Undiluted	Neg.	Neg.		
3	1:10	32.8	32.6		Positive
3	1:100	35.5	36.0		
3	Undiluted	37.0	33.0		
3	1:10	30.6	32.8		Second preparation
3	1:100	35.5	38		
4	Undiluted	>40	Neg.		
4	1:10	32.9	32.5		Positive
4	1:100	36.2	35.9		
4	Undiluted	>40	38.0		
4	1:10	>40	32.7		Second preparation
4	1:100	Neg.	36.6		
5	Undiluted	Neg.	Neg.		
5	1:10	Neg.	Neg.		
5	1:100	Neg.	Neg.		
5	Undiluted	Neg.	Neg.		
5	1:10	Neg.	Neg.		Second preparation
5	1:100	Neg.	Neg.		
6	Undiluted	Neg.	Neg.		
6	1:10	Neg.	Neg.		
6	1:100	Neg.	Neg.		
7	Undiluted	36.8	36.5		
7	1:10	37.8	36.7		Positive
7	1:100	39.0	36.8		
8	Undiluted	35.7	NA		No spleen tissue
8	1:10	34.0	NA		Positive
8	1:100	37.2	NA		
9	Undiluted	Neg.	Neg.		
9	1:10	Neg.	Neg.		
9	1:100	Neg.	Neg.		
10	Undiluted	Neg.	Neg.		
10	1:10	Neg.	Neg.		
10	1:100	Neg.	Neg.		

Puppy no.	Dilutions	PCR (Cp value)		Additional tissue analyzed	Remarks
		Liver/lung	Spleen/kidney		
11	Undiluted	Neg.	35.2		
11	1:10	Neg.	>40		Positive
11	1:100	Neg.	37.7		
12	Undiluted	37.1	>40		
12	1:10	>40	39.4		Positive
12	1:100	>40	Neg.		
13	Undiluted	33.1	>40		
13	1:10	34.8	36.9		Positive
13	1:100	39.5	>40		
14	Undiluted	Neg.	Neg.		
14	1:10	Neg.	Neg.		
14	1:100	Neg.	Neg.		
15	Undiluted	32.8	38.5		
15	1:10	35.7	>40		Positive
15	1:100	Neg.	Neg.		
16	Undiluted	34.5	38.5		
16	1:10	38.9	Neg.		Positive
16	1:100	Neg.	Neg.		
17	Undiluted	29.6	36.6		
17	1:10	33.6	>40		Positive
17	1:100	36.9	Neg.		
18	Undiluted	29.7	Neg.		
18	1:10	31.3	Neg.		Positive
18	1:100	35.6	Neg.		
19	Undiluted	Neg.	Neg.	Heart: 37.8	
19	1:10	Neg.	Neg.	Heart: 38.2	Positive
19	1:100	Neg.	Neg.	Heart: Neg.	
19	Undiluted	Neg.	Neg.	Heart: >40	Second preparation
19	1:10	Neg.	Neg.	Heart: Neg.	Negative
19	1:100	Neg.	Neg.	Heart: Neg.	
20	Undiluted	Neg.	Neg.		
20	1:10	Neg.	Neg.		
20	1:100	Neg.	Neg.		
21	Undiluted	Neg.	Neg.		
21	1:10	Neg.	Neg.		
21	1:100	Neg.	Neg.		
22	Undiluted	Neg.	Neg.		
22	1:10	Neg.	Neg.		
22	1:100	Neg.	Neg.		
23	Undiluted	Neg.	Neg.		
23	1:10	Neg.	Neg.		
23	1:100	Neg.	Neg.		
24	Undiluted	Neg.	Neg.		
24	1:10	Neg.	Neg.		
24	1:100	Neg.	Neg.		

Puppy no.	Dilutions	PCR (Cp value)		Additional tissue analyzed	Remarks
		Liver/lung	Spleen/kidney		
25	Undiluted	Neg.	Neg.		
25	1:10	Neg.	Neg.		
25	1:100	Neg.	Neg.		
26	Undiluted	Neg.	Neg.		
26	1:10	Neg.	Neg.		
26	1:100	Neg.	Neg.		
27	Undiluted	Neg.	Neg.		
27	1:10	Neg.	Neg.		
27	1:100	Neg.	Neg.		
28	Undiluted	Neg.	Neg.		
28	1:10	Neg.	Neg.		
28	1:100	Neg.	Neg.		
29	Undiluted	Neg.	Neg.		
29	1:10	Neg.	Neg.		
29	1:100	Neg.	Neg.		
30	Undiluted	Neg.	Neg.		
30	1:10	Neg.	Neg.		
30	1:100	Neg.	Neg.		
31	Undiluted	Neg.	Neg.		
31	1:10	Neg.	Neg.		
31	1:100	Neg.	Neg.		
32	Undiluted	Neg.	Neg.		
32	1:10	Neg.	Neg.		
32	1:100	Neg.	Neg.		
33	Undiluted	Neg.	Neg.		
33	1:10	Neg.	Neg.		
33	1:100	Neg.	Neg.		
34	Undiluted	>40	Neg.		
34	1:10	Neg.	Neg.		
34	1:100	Neg.	Neg.		
35	Undiluted	Neg.	Neg.		
35	1:10	Neg.	Neg.		
35	1:100	Neg.	Neg.		
36	Undiluted	Neg.	Neg.		
36	1:10	Neg.	Neg.		
36	1:100	Neg.	Neg.		
37	Undiluted	Neg.	Neg.		
37	1:10	Neg.	Neg.		
37	1:100	Neg.	Neg.		
38	Undiluted	Neg.	Neg.		
38	1:10	Neg.	Neg.		
38	1:100	Neg.	Neg.		
39	Undiluted	Neg.	Neg.		
39	1:10	Neg.	Neg.		
39	1:100	Neg.	Neg.		

Puppy no.	Dilutions	PCR (Cp value)		Additional tissue analyzed	Remarks
		Liver/lung	Spleen/kidney		
40	Undiluted	Neg.	Neg.		
40	1:10	Neg.	Neg.		
40	1:100	Neg.	Neg.		
41	Undiluted	Neg.	Neg.		
41	1:10	Neg.	Neg.		
41	1:100	Neg.	Neg.		
42	Undiluted	>40	Neg.		
42	1:10	Neg.	Neg.		
42	1:100	Neg.	Neg.		
43	Undiluted	Neg.	Neg.		
43	1:10	Neg.	Neg.		
43	1:100	Neg.	Neg.		
44	Undiluted	Neg.	Neg.		
44	1:10	Neg.	Neg.		
44	1:100	Neg.	Neg.		
45	Undiluted	Neg.	Neg.		
45	1:10	Neg.	Neg.		
45	1:100	Neg.	Neg.		
46	Undiluted	Neg.	Neg.		
46	1:10	Neg.	>40		
46	1:100	Neg.	Neg.		
47	Undiluted	Neg.	Neg.		
47	1:10	Neg.	Neg.		
47	1:100	Neg.	Neg.		
48	Undiluted	Neg.	Neg.		
48	1:10	Neg.	Neg.		
48	1:100	Neg.	Neg.		
49	Undiluted	Neg.	Neg.		
49	1:10	Neg.	Neg.		
49	1:100	Neg.	Neg.		
50	Undiluted	Neg.	Neg.		
50	1:10	Neg.	Neg.		
50	1:100	Neg.	Neg.		
51	Undiluted	Neg.	Neg.		
51	1:10	Neg.	Neg.		
51	1:100	Neg.	Neg.		
52	Undiluted	Neg.	Neg.		
52	1:10	Neg.	Neg.		
52	1:100	Neg.	Neg.		
53	Undiluted	Neg.	Neg.		
53	1:10	Neg.	Neg.		
53	1:100	Neg.	Neg.		
54	Undiluted	Neg.	Neg.		
54	1:10	Neg.	Neg.		
54	1:100	Neg.	Neg.		

Puppy no.	Dilutions	PCR (Cp value)		Additional tissue analyzed	Remarks
		Liver/lung	Spleen/kidney		
55	Undiluted	Neg.	Neg.		
55	1:10	Neg.	Neg.		
55	1:100	Neg.	Neg.		
56	Undiluted	Neg.	Neg.		
56	1:10	Neg.	Neg.		
56	1:100	Neg.	Neg.		
57	Undiluted	Neg.	Neg.		
57	1:10	Neg.	Neg.		
57	1:100	Neg.	Neg.		

NA: Tissues not available

Neg.: Negative sample.

*Cp > 40 was considered positive though indicating a minimal amount of nuclei acid.
